# Retrospective investigation of IgM antibodies against Zika virus in serum from febrile patients in Mozambique, 2009–2015

**DOI:** 10.1186/s13104-019-4511-x

**Published:** 2019-07-31

**Authors:** Imelda Rogério Chelene, Sadia Ali, Flora Inalda Mula, Argentina Felisbela Muianga, Vanessa Onofre Monteiro, John Oludele, Inocêncio Salvador Chongo, Américo José, Nádia Alves Amade, Virgílio Santo António, Eduardo Samo Gudo

**Affiliations:** 1 Instituto Nacional de Saúde, Vila de Marracuene, EN1, Parcela nº3943, Maputo, Mozambique; 2 Centro de Colaboração em Saúde, Rua Damião de Gois, Maputo, Mozambique

**Keywords:** Zika, Mozambique, Febrile patient

## Abstract

**Objective:**

Zika virus (ZIKV) has emerged as an important health problem worldwide. The aim of this study was to investigate the occurrence, geographical distribution and trend of immunoglobulin M (IgM) antibodies against ZIKV between 2009 and 2015 in Mozambique.

**Results:**

The median age of participants was 3 years [interquartile range (IQR): 1.0–6.0 years)] and 56.5% (480/850) of them were male. Of the 850 samples, 42 (4.9%) were positive for IgM antibodies against ZIKV. Positive samples were found in 9 provinces of the country. Frequency of IgM antibodies against ZIKV was slightly higher in patients aged 5–9 years old, and in the north region of the country.

## Introduction

Zika virus (ZIKV) belongs to the genus *Flavivirus*, family *Flaviviridae* [1, 2]. The virus is mostly transmitted through the bite of infected *Aedes* mosquitoes. The primary *Aedes* species vector of Zika virus worldwide is *Ae. aegypti* and recent research found that *Ae. aegypti* is the most abundant *Aedes* species in Mozambique [1, 3]. Transmission can also occur from mother to child during pregnancy or spread through sexual contact and blood transfusion [1, 4].

Most of ZIKV infections are asymptomatic and only 20–25% of the infected people develop a mild and self-limited illness. Zika viral infection may present the following symptoms: fever, joint pain, rash and conjunctivitis (at a lesser frequency), retro-orbital pain, headache, myalgia, edema, and vomiting [1, 5]. In few patients, ZIKV can cause severe disease, namely, neurological diseases, such as Guillain–Barré syndrome in infected adults and microcephaly in infants born to ZIKV-infected women [1, 2, 4]. For the first 60 years, ZIKV was confined to an equatorial zone across Africa and Asia, however, over the last decade the virus has experienced an unprecedented global spread to affect other regions, followed by an explosive spread in South America in 2016 [1, 4]. In the sub-Saharan Africa region, sporadic cases of ZIKV were reported in several countries since its discovery [6, 7]. Neutralizing antibodies against ZIKV were found for the first time in Mozambique 1957 [8] and since then, no other study was conducted and the virus remained mostly neglected in the country. As a consequence, most of the recent literature consistently excluded Mozambique from the list of countries with potential circulation of ZIKV [4, 9, 10]. Due to the potential risk of current circulation of the virus, there is an urgency to investigate its occurrence in Mozambique. In this context, we conducted this investigation aiming at retrospectively investigate the occurrence, geographical distribution and trend of IgM antibodies against ZIKV in samples from a serum bank of measles and rubella surveillance collected between 2009 and 2015 in Mozambique.

## Main text

### Methods

#### Study design, settings, and samples

In this study, we retrieved 850 samples from the serum bank stored at the Serology Laboratory of the National Institute of Health in Mozambique. These samples were collected as part of the routine case-based surveillance for measles in Mozambique across multiple districts in the country. In Mozambique, measles surveillance follows WHO guidelines and are eligible for measles surveillance patient with fever and one of the following symptoms: rash and cough, coryza or conjunctivitis [11]. Only samples from patients recruited between 2009 and 2015 with measles and rubella negative results were eligible. These samples were tested for ZIKV because fever and rash are common symptoms of infection by ZIKV [5, 12]. All samples with insufficient serum volume, inappropriate labeling, without demographic data in the database or deteriorated were excluded.

#### Laboratory testing

Serum samples (n = 850) were screened for Zika antibodies (IgM) using commercially available ELISA kit (Euroimmun Lübeck, Germany) at Virus Isolation Laboratory (LIV), in Maputo, Mozambique following the manufacturer’s instructions.

#### Statistical analysis

For each sample, demographic information was retrieved from the electronic database of the measles surveillance available at the Serology laboratory of the National Institute of Health which was developed using Epi Info 3 version 3.5.1. The variables retrieved from this database were: age, gender, district, province, date of onset, date of specimen collection and year.

Data was analyzed using the statistical software package SPSS 20.0. A p-value < 0.05 was considered statistically significant.

### Results

#### Demographical characteristics of participants and frequency of antibodies against ZIKV

The median age of participants was 3.0 years [interquartile range (IQR): 1.0–6.0 years)] and 56.5% (480/850) of them were male. In terms of age distribution, the most frequent age category was 0–1 years old (37.4%, 318/850), followed by age category of 2–4 years old (28.2%, 240/850) and age category of 5-9 years old (21.8%, 186/850) (see Table 1). Frequency of participants from central region of Mozambique was 41.9% (356/850), followed by participants from north (35.4%, 301/850) and southern region (22.7%, 193/850).

**Table 1:** Demographic characteristics among cases with IgM anti-ZKV cases

	Suspected cases reported (n)	Zika IgM	Proportion of IgM+ (95% CI), %	Proportion ratio (95% CI)	p-value
Total	850	42	4.9 (3.5–6.6)		
Sex					0.299
Male	480	27	5.6 (3.7–7.9)	1	
Female	369	15	4.1 (2.1–6.2)	3.1 (2.6–3.7)	
Age					
Median (IQR)	3.0 (1.0–6.0)	3.0 (1.0–5.0)			
Age (years)					0.521
0–1	318	7	2.2 (16.2–20.8)	1	
2–4	240	14	5.8 (25.4–31.5)	1.45 (0.25–8.16)	
5–9	186	14	7.5 (18.9–24.9)	0.71 (0.15–3.28)	
10–14	67	3	4.5 (6.1–9.8)	0.59 (0.12–2.74)	
≥ 15	39	4	4.6 (3.3–6.3)	0.54 (0. 11–2.55)	
Regions					0.087
North	301	20	6.6 (10.2–17.0)	1	
Central	356	17	4.7 (3.9–9.0)	0.69 (0.81–1.32)	
South	193	5	2.5 (8.5–17.4)	1.89 (0.61–4.32)	
Year of onset					0.356
2009	62	2	3.22 (5.7–9.1)	1.61 (0.35–7.29)	
2010	121	8	6.6 (12.0–16.6)	0.76 (0.31–1.86)	
2011	84	8	9.5 (8.0–12.0)	0.59 (0.23–1.52)	
2012	88	6	6.8 (8.4–12.6)	0.73 (0.27–1.97)	
2013	142	4	2.8 (14.3–19.3)	1.85 (0.6–5.75)	
2014	78	1	1.2 (7.2–11.2)	4.14 (0.53–32.0)	
2015	275	13	4.7 (29.3–35.4)	1	

*IgM+* measles suspected cases with positive result for IgM anti-Zika; *CI* confidence interval

Serum samples were mostly from 2015 (275/850; 32.4%), followed by 2013 (142/850; 16.7%) and 2010 (121/850; 14.2%).

**Fig. 1:**
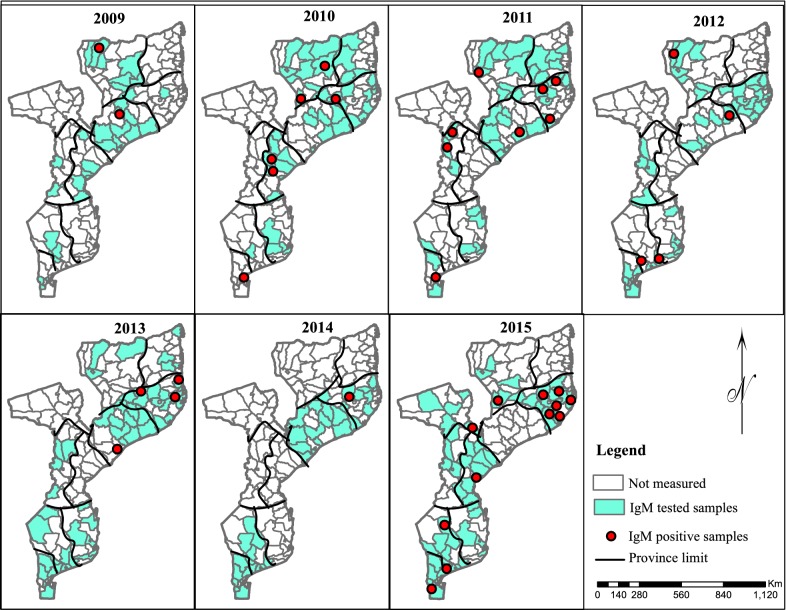
Distribution of IgM anti-ZIKV cases

Of the 850 samples, 42 (4.9%) were positive for IgM antibodies against ZIKV. In terms of geographical distribution, IgM antibodies against ZIKV were detected in 9 provinces of the country (see Fig. 1). No IgM antibodies against ZIKV was found in samples from Cabo Delgado, and Inhambane provinces.

#### Characteristics of IgM positive patients

The median age of IgM-positive participants was 3.0 years [IQR: 1.0–5.0 years)] and 64.3% (27/42) were male. Frequency of IgM antibodies against ZIKV was higher in patients aged 5–9 years old (7.5; 95% CI 18.9–24.9) (see Table 1).

Frequency of IgM anti-ZIKV was higher in north region. No trend across years was noted, but the highest frequency of IgM anti-ZIKV was found in 2011 (see Table 1).

### Discussion

In this study, we retrospectively found serological evidence of ZIKV in samples retrieved from the serum bank of febrile patients enrolled into measles surveillance in Mozambique between 2009 and 2015. This suggests that Mozambicans were silently exposed to the virus over the last decades. However, if ZIKV is circulating in the country since 1957 when it was for the first time reported in the country or if this represent a more recent re introduction is yet to be determined.

Our results suggest that cases of ZIKV are misdiagnosed and treated as measles, rubella, or other common acute febrile illness. These findings are important because: (i) recent publications addressing global risk of ZIKV exclude Mozambique from the list of countries with current presence of ZIKV infections [4, 9, 10] and (ii) ZIKV has been heavily neglected by the local authorities in Mozambique.

Frequency of IgM anti-ZIKV was higher in the north region of the country, which corroborates with findings from recent studies conducted in Mozambique showing that DENV and CHIKV occurs in the north and center of the country [13, 14]. Moreover, an outbreak of dengue virus serotype 2 (DENV-2) was reported in 2014 in Nampula and Pemba cities, situated in northern Mozambique, and a prospective surveillance, found that DENV-2 have become endemic in northern Mozambique [15]. These findings indicates that arboviruses are co-circulating in north of the country, suggesting that the northern region of the country might be a hotspot for occurrence of arboviruses in Mozambique.

Cross reaction of ZIKV antibodies with other flavivirus antibodies is known to occur [16]. However, the EUROIMMUNE reagents used in this study are claimed to be sensitive and specific [16–18].

This is the first serological investigation of ZIKV in Mozambique since 1957 and found anti-ZIKV antibodies in serum of febrile patients in 9 provinces of the country from 2009 to 2015, suggesting that Mozambicans were silently exposed to the virus. Data from this study suggests that ZIKV should be considered in the differential diagnosis of fever. We recommend that a surveillance system for ZIKV should be established to monitor occurrence of ZIKV in the country.

## Limitations

This study is based on serologic test using commercial kit ELISA, other approaches that include molecular tests, immunofluorescence or neutralizing assay will be of great relevance for next interventions.

## Data Availability

The datasets used and/or analyzed during the current study are available from the corresponding author on reasonable request.

## Abbreviations

ZIKV: Zika virus
IgM: immunoglobulin M
ELISA: enzyme linked immunosorbent assay
